# PD-L1 Induction by Cancer-Associated Fibroblast-Derived Factors in Lung Adenocarcinoma Cells

**DOI:** 10.3390/cancers11091257

**Published:** 2019-08-27

**Authors:** Chihiro Inoue, Yasuhiro Miki, Ryoko Saito, Shuko Hata, Jiro Abe, Ikuro Sato, Yoshinori Okada, Hironobu Sasano

**Affiliations:** 1 Department of Anatomic Pathology, Tohoku University Graduate School of Medicine, Sendai, Miyagi 981-8575, Japan; 2 Department of Disaster Obstetrics and Gynecology, International Research Institute of Disaster Science, Tohoku University, Sendai, Miyagi 980-8573, Japan; 3 Division of Pathology, Faculty of Medicine, Tohoku Medical and Pharmaceutical University, Sendai, Miyagi 981-8558, Japan; 4 Department of Thoracic Surgery, Miyagi Cancer Centre, Natori, Miyagi 981-1293, Japan; 5 Department of Pathology, Miyagi Cancer Centre, Natori, Miyagi 981-1293, Japan; 6 Department of Thoracic Surgery, Institute of Development, Aging and Cancer, Tohoku University, Sendai, Miyagi 981-8575, Japan

**Keywords:** cancer associated fibroblasts, tumor microenvironment, α-smooth muscle actin, programmed death ligand 1, lung adenocarcinoma

## Abstract

Cancer-associated fibroblasts (CAFs) exert various effects upon biological behaviours of cancer. In this study, we examined the correlation of CAFs with the intra-tumoural immune system in the lung adenocarcinoma microenvironment. We studied 27 and 113 cases of lung adenocarcinoma tentatively as Cohorts 1 and 2, respectively. The patients in Cohort 1 received epidermal growth factor receptor-tyrosine kinase inhibitor (EGFR-TKI) for recurrent lung adenocarcinoma. α-smooth muscle actin (α-SMA), a surrogate marker for CAFs, was examined by immunohistochemistry. We then examined the effects of CAFs isolated from lung cancer tissues on programmed death ligand 1 (PD-L1) expression in lung adenocarcinoma cell lines. No significant associations were detected between α-SMA status and the ratios of CD8/CD4 and Foxp3/CD8 in Cohort 1. However, α-SMA status was significantly associated with PD-L1 status in both Cohorts 1 and 2. Conditioned medium of CAFs significantly induced PD-L1 expression in lung adenocarcinoma cell lines, A549, PC-9, and H1975. Among the cytokines examined by antibody array, C-X-C motif chemokine ligand 2 (CXCL2) increased PD-L1 mRNA expression in these cell lines. CXCL2 is therefore considered to have a potential to induce PD-L1 expression in lung adenocarcinoma cells as a result of an interaction between carcinoma cells and CAFs. These findings did firstly demonstrate that CAFs indirectly influenced tumour immunity through increasing PD-L1 expression in lung adenocarcinoma cells.

## 1. Introduction

Lung adenocarcinoma cells have been well known to interact with various compartments of tissue microenvironment in cancer tissue, including immune cells, microvessels, and fibroblasts. Activated fibroblasts within cancer stroma are termed cancer-associated fibroblasts (CAFs) and have been known to be associated with cancer growth, invasion, migration, metastasis, and therapeutic resistance through secretion of various soluble factors, including cytokines, chemokines, growth factors, and exosomes [1,2,3,4,5,6]. CAFs have also been reported to influence tumour immunity in various human malignancies [1,2,7,8,9]. In addition, lymphocytes play important roles in tumour immunity; for example, CD8+ cytotoxic T-cells damage tumour cells, whereas CD4+Foxp3+ regulatory T-cells suppress the function of these cells [10,11].

The analysis of the interaction between carcinoma cells and their tissue microenvironment components has attracted enormous attention. In particular, immune checkpoints, namely programmed death 1 (PD-1) and PD ligand 1 (PD-L1), have been known to play a pivotal role in the prevention of autoimmunity, but in cancer, they are utilized to evade the tumour immune response of the host. Several immune checkpoint inhibitors have been clinically approved for the treatment of different cancers, including non-small cell lung cancer (NSCLC). Anti-PD-1/PD-L1 inhibitors have been administered to patients with NSCLC in advanced stages, and the status of PD-L1 immunoreactivity in these carcinoma cells has also been reported to be significantly associated not only with the therapeutic effects of anti-PD-1/PD-L1 inhibitors, but also with eventual clinical outcome of these patients [12,13,14,15,16]. PD-L1 in carcinoma cells has been reported to be induced by exposure to inflammatory cytokines, including IFN-γ, and activation of oncogenic pathways, such as PI3K, STAT3, MEK, and Akt-mTOR [17,18,19,20,21]. CAFs also secrete inflammatory cytokines and growth factors, which subsequently activate oncogenic pathways in carcinoma cells. Therefore, in this study, we hypothesized that CAFs could indirectly suppress tumour immunity via induction of PD-L1 expression in carcinoma cells.

We first examined the influence of CAFs on PD-L1 expression in carcinoma cells in lung adenocarcinoma tissue. We evaluated the correlation between immunoreactivity of α-smooth muscle actin (α-SMA), a well-known marker of CAFs, and subpopulations of tumour-infiltrating lymphocytes determined by CD3, CD4, CD8, and Foxp3, in 27 lung adenocarcinoma tissues. We then immunolocalized α-SMA in 113 lung adenocarcinoma cases and examined the effect of isolated CAFs from lung adenocarcinoma tissues on expression of PD-L1 in lung adenocarcinoma cell lines in vitro. Cytokines released from CAFs were detected using a cytokine array, and the effects of cytokines on PD-L1 expression in lung adenocarcinoma cell lines were also studied.

## 2. Results

### 2.1. α-SMA Status in Stromal Area of Lung Cancer Was Significantly Associated with PD-L1 Status in Carcinoma Cells and Adverse Clinical Outcome of the Patients

**Figure 1:**
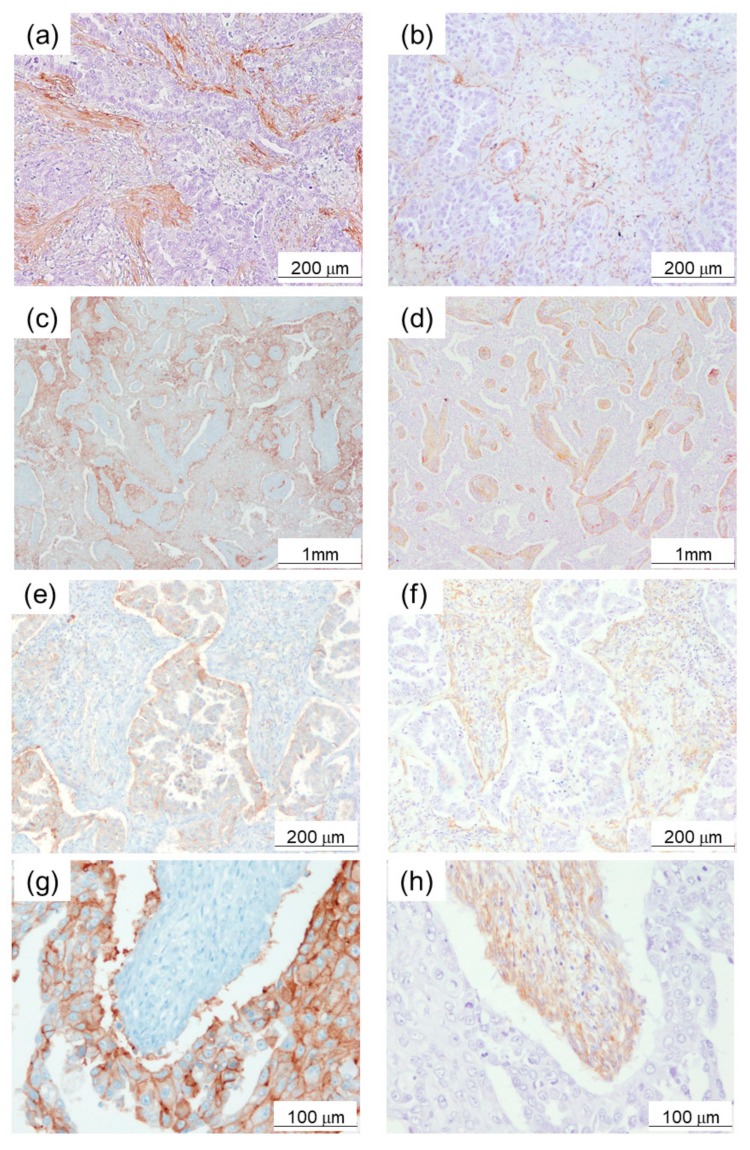
Expression of α-smooth muscle actin (α-SMA) in lung adenocarcinoma tissue samples. (**a**) High expression example (≥60%). (**b**) Low expression example (<60%). (**c**), (**e**), (**g**) Programmed death ligand 1 (PD-L1), and (**d**), (**f**), (**h**) α-SMA immunoreactivity in serial tissue sections of mirror images of lung adenocarcinoma. High PD-L1 immunoreactivity was detected in adenocarcinoma cells adjacent to α-SMA-positive stroma.

α-SMA immunoreactivity was detected in the cytoplasm of fibroblast-like stromal cells in lung adenocarcinoma tissues (Figure 1a,b). The median value of the percentage of the α-SMA-positive area to the stromal area was 60% in Cohort 1. The patients were then tentatively classified into two groups according to the median value of intratumoural α-SMA status: High (≥60%), and low expression group (<60%) (Figure 1a,b). 

**Figure 2:**
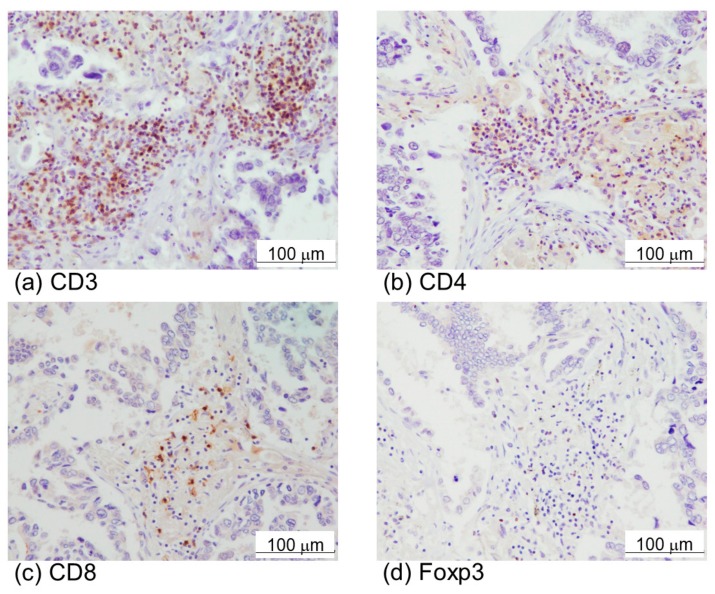
Immunohistochemistry of immune cell markers, CD3 (**a**), CD4 (**b**), CD8 (**c**), and Foxp3 (**d**) in lung adenocarcinoma.

**Figure 3:**
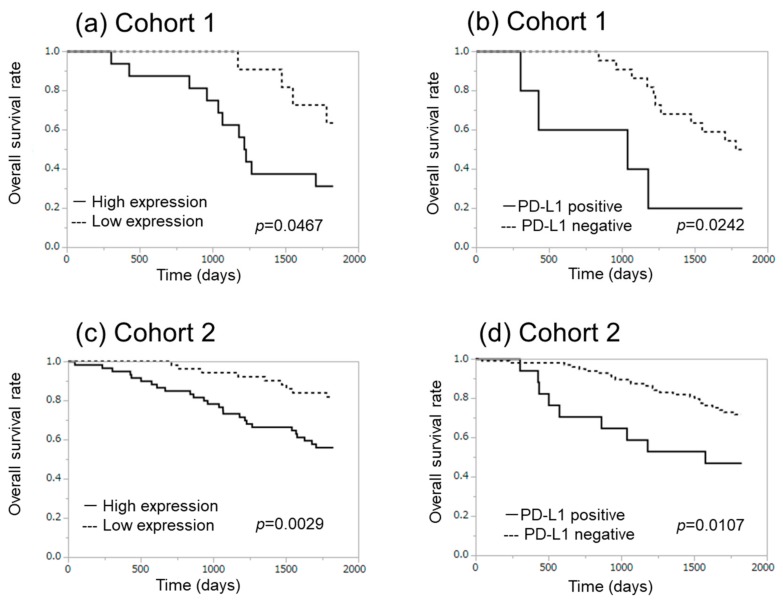
Correlation between overall survival and α-SMA ((**a**) Cohort 1, (**c**) Cohort 2) and PD-L1 ((**b**) Cohort 1, (**d**) Cohort 2) expression in lung adenocarcinoma cases.

High expression of α-SMA was also significantly associated with advanced pathological stage (Table 1). In addition, high expression of α-SMA tended to be correlated with higher pN and PD-L1 positivity. However, α-SMA was by no means significantly associated with the effects of epidermal growth factor receptor-tyrosine kinase inhibitor (EGFR-TKI), nor with the status of intratumoural infiltrating lymphocytes in Cohort 1. Representative CD3, CD4, CD8, and Foxp3 immunoreactivity was illustrated in Figure 2a–d. There were also no significant associations between α-SMA status and the ratio of CD8/CD4 and Foxp3/CD8 (Table 1). The Kaplan–Meir analysis revealed that the five-year overall survival with high and low α-SMA groups was 31.3% and 63.6%, respectively (χ^2^ = 4.93, *p*-value = 0.0467) in Cohort 1 (Figure 3a). The five-year overall survival with positive and negative PD-L1 was 20.0% and 50.0%, respectively (χ^2^ = 5.08, *p*-value = 0.0242), in Cohort 1 (Figure 3b).

In Cohort 2, the median value of the Brinkman index was significantly higher in the high α-SMA group (Table 2). α-SMA status was also significantly associated with smoking history, pStage, and PD-L1 status of adenocarcinoma cells in Cohort 2 (Table 2). High α-SMA status was significantly associated with gender of the patients (male > female), smoking history (smoker > non-smoker), higher Brinkman index, advanced clinical stage, and PD-L1 immunoreactivity in Cohort 2 (Table 2). The Kaplan–Meir plots also did demonstrate that the five-year overall survival with high and low α-SMA groups was 56.7% and 83.0%, respectively (χ^2^ = 8.84, *p*-value = 0.0029; Figure 3c). The five-year overall survival with positive and negative PD-L1 in carcinoma cells was 47.1% and 72.9%, respectively (χ^2^ = 6.51, *p*-value = 0.0107) in Cohort 2 (Figure 3d). Multivariate analysis demonstrated that the status of α-SMA was not necessarily an independent prognostic factor.

Of particular interest, all PD-L1-positive cases corresponded to the high α-SMA group. Representative findings of α-SMA and PD-L1 immunohistochemistry in serial tissue sections with mirror images were illustrated in Figure 1c–h. High PD-L1 immunoreactivity was detected in adenocarcinoma cells adjacent to α-SMA-positive stroma.

### 2.2. Conditioned Medium of CAFs Significantly Increased PD-L1 Expression at Both mRNA and Protein Levels

**Figure 4:**
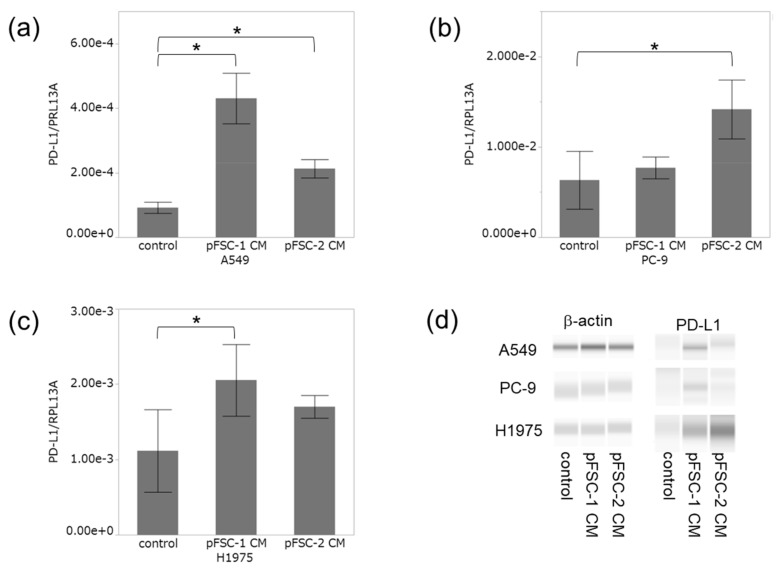
Effect of conditioned medium (CM) derived from cancer-associated fibroblasts (CAFs) on PD-L1 expression in lung cancer cell lines. Effect of CM from pFSC-1 and -2 on PD-L1 mRNA level in A549 (**a**), PC-9 (**b**), and H1975 (**c**). Data were presented as means ± SD from three independent experiments. * *p* < 0.05 vs. control. (**d**) Protein level of PD-L1 treated with CM in A549, PC-9, and H1975.

pFSC-1 and pFSC-2 were classified as CAFs because α-SMA was detected in their cytoplasm using immunocytochemistry (Supplementary Figure S1). Conditioned medium (CM) collected from pFSC-1 significantly increased PD-L1 mRNA levels in A549 and H1975 cells (Figure 4a,c), and pFSC-2 conditioned medium also significantly increased PD-L1 mRNA levels in A549 and PC-9 cells (Figure 4b). PD-L1 immunoreactivities were increased by pFSC-1 conditioned medium in A549 and H1975, and by pFSC-2 in all cell lines examined (Figure 4d). Electropherograms and lane views of PD-L1 were also demonstrated in Supplementary Figure S2. It is well known that PD-L1 is affected by glycosylation [22]. Therefore, heterogeneous expression of PD-L1 protein around 45 kDa is detected by immunoassay (Supplementary Figure S2). Furthermore, in this study, more large sizes (approximately 50–70 kDa) of PD-L1 immunoreactivity were detected in lung cancer cell lines (Supplementary Figure S2). Although large sizes in PD-L1 immunoassay have been reported [23], their significance is unclear. Therefore, PD-L1 expression in approximately 45 kDa was evaluated in this study (Figure 4d).

### 2.3. Profiles of Cytokines Secreted from CAFs Were Altered by Co-Culture with Adenocarcinoma Cells

**Figure 5:**
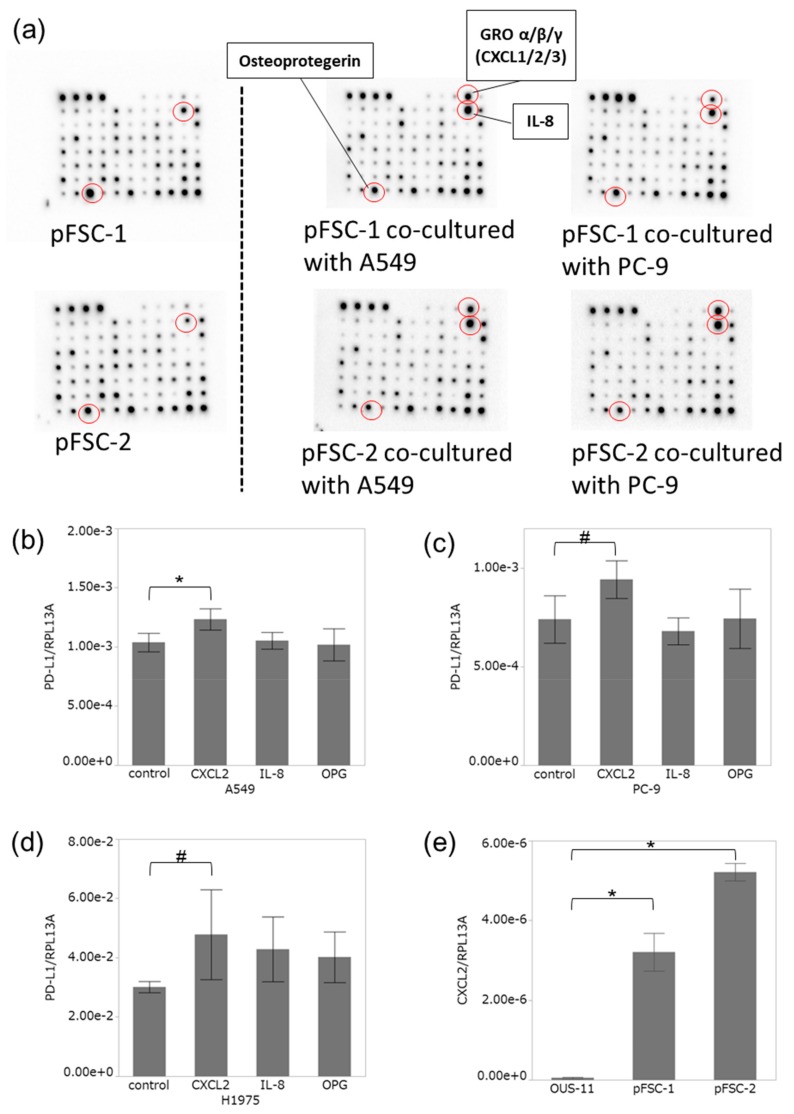
Cytokine antibody array image and effect of cytokines on PD-L1 expression in lung cancer cell lines. (**a**) The array image showing osteoprotegerin (OPG), growth related oncogene (GRO) -α/β/γ (C-X-C motif chemokine ligand: CXCL1/2/3), and IL-8 in pFCS-1 (upper) and -2 (lower) CM with/without co-culture. Effect of CXCL2, IL-8, and OPG on PD-L1 mRNA level in A549 (**b**), PC-9 (**c**), and H1975 (**d**). (b) A549: CXCL2 100 ng/mL, IL-8 100 ng/mL, OPG 100 ng/mL; (c) PC-9 and (d) H1975: CXCL2 10 ng/mL, IL-8 10 ng/mL, OPG 50 ng/mL. (**e**) CXCL2 mRNA level in CAFs (pFSC-1 and pFSC-2) and normal lung fibroblasts (OUS-11). Data were presented as means ± SD from three independent experiments. * *p* < 0.05 vs. control. # 0.05 ≤ *p* < 0.1 vs. control.

Results of cytokine array analysis did reveal that both IL-8 and osteoprotegerin (OPG) were markedly detected in conditioned medium collected from both pFSC-1 and pFSC-2. Growth related oncogene (GRO) -α/β/γ (C-X-C motif chemokine ligand: CXCL1/2/3) and IL-8 in conditioned medium were also increased, and OPG was decreased by the co-culture with A549 and PC-9 cells (Figure 5a), whereas GROα (CXCL1) was not influenced by co-culture. We then examined the effects of IL-8 and OPG on PD-L1 expression in lung adenocarcinoma cell lines. In this study, we particularly focused on CXCL2, a chemokine released from CAFs in lung adenocarcinoma cells. CXCL2 was also detected in CM of both pFSC-1 and -2 in cytokine array analysis.

### 2.4. CXCL2 Increased PD-L1 mRNA in Adenocarcinoma Cells

We studied the influence of CXCL2, IL-8, and OPG on PD-L1 mRNA level in A549, PC-9, and H1975. CXCL2 (100 ng/mL) significantly increased PD-L1 mRNA level in A549 (Figure 5b), and a similar trend was detected in PC-9 and H1975 cells with CXCL2 (10 ng/mL) administration (Figure 5c,d). Both IL-8 and OPG did not influence PD-L1 mRNA levels in A549, PC-9, and H1975.

We then examined CXCL2 mRNA level in CAFs and normal fibroblasts. In this examination, we employed normal lung fibroblasts, OUS-11. OUS-11 had no immunoreactivity of α-SMA (Supplementary Figure S1). CXCL2 mRNA level was significantly higher in CAFs, pFSC-1 and pFSC-2, than in OUS-11 (Figure 5e).

## 3. Discussion

CAFs were reported to interact with carcinoma cells, and influence their biological behaviour in vitro. Several markers, including α-SMA, periostin, PDGFRα, PDGFRβ, podoplanin, and fibroblast activation protein (FAP), have been employed to characterize CAFs, although their roles in CAFs have remained unknown. Among these surrogate markers, α-SMA is the most commonly used CAF marker, and Horie et al. [3] reported that primary culture of CAFs isolated from non-small cell lung cancer (NSCLC) express more α-SMA than normal fibroblasts. Meta-analysis on immunohistochemical study of α-SMA in stromal areas of cancer tissue demonstrated that a higher status of α-SMA was significantly associated with poor overall survival [24]. Therefore, in this study, we employed α-SMA as a surrogate marker for CAFs in lung adenocarcinoma tissues. 

Two previous studies were reported on the correlation between α-SMA status in stromal area and clinicopathological characteristics of the patients with NSCLC. Chen et al. [25] reported that intratumoural α-SMA status was significantly associated with lower three-year survival in 78 NSCLC patients with clinical stages I to III. They also evaluated the percentage of positive α-SMA stained areas in cancer stromal area in 10 high-power fields randomly selected in each slide. However, another study reported that α-SMA did not influence prognosis of the patients with NSCLC, after examining 633 tissue-microarray specimens (0.6 mm core) [26]. In this study, we employed sectioned specimens from 113 lung adenocarcinoma cases. We demonstrated that the status of α-SMA in cancer stromal area was significantly associated with an advanced pathological stage and poor five-year survival rate of the patients with lung adenocarcinoma. Heterogeneity of distribution of CAFs in lung cancer tissues could result in a discrepancy of these interpretations about the roles of α-SMA-positive CAFs in a cancer tissue microenvironment. The results in our present study also suggested that CAFs in lung adenocarcinoma tissue could promote cancer progression. CAFs are also known to regulate biological behaviour of carcinoma cells, such as growth, invasion, metastasis, and therapeutic resistance. In this study, we confirmed that pFSC-1 and pFSC-2 promoted proliferation and migration of lung adenocarcinoma cells (Supplementary Figures S3 and S4). CAFs, which were α-SMA-positive stroma, may have contributed to growth and/or metastasis of lung adenocarcinoma of the patients in this study. 

We also focused on immune cells related to CAFs in lung adenocarcinoma tissue because CAFs can directly modulate activity of tumour immunity [1,2,7,8,9]. Contrary to our expectations, α-SMA-positive CAFs in lung adenocarcinoma were by no means correlated with lymphocyte subtypes evaluated by CD3, CD4, CD8, and Foxp3 immunohistochemistry. Nazareth MR et al. reported that while some CAFs promoted activation of T-cells, other CAFs suppressed them [8]. Immune-suppressive CAFs and immune-promoting CAFs may have been intermingled in a lung adenocarcinoma tissue in this study. Next, we focused on PD-L1 expression in the interaction between CAFs and carcinoma cells. The report about hepatocellular carcinoma demonstrated that IL-6, a CAFs-derived factor, was also reported to play an important role in the functions of dendritic cells through STAT3 signalling [27]. We hypothesized that CAFs might influence tumour immunity through non-lymphocyte cells, including cancer cells. As a result, the α-SMA status in the cancer tissue microenvironment was significantly associated with PD-L1 status in lung adenocarcinoma cells. PD-L1 is well known to be induced by several cytokines, such as IFN-γ, TNF-α, IL-4, and IL-10 [14,17,28]. Therefore, PD-L1 in lung carcinoma cells might be directly induced by CAFs-derived cytokines in lung adenocarcinoma. Hence, we then examined the effects of soluble factors derived from primary culture of CAFs on PD-L1 expression in lung adenocarcinoma cells in vitro. Addition of conditioned medium collected from α-SMA-positive CAFs increased PD-L1 mRNA and protein in A549, PC-9, and H1975 in this study. 

We examined the cytokine profiles derived from primary culture of CAFs by using cytokine array, and then evaluated the effects of CXCL2, IL-8, and OPG on PD-L1 expression in A549, PC-9, and H1975. PD-L1 mRNA was significantly increased by treatment with CXCL2 alone. CXCL2 belongs to the CXCL family of chemokines bearing the ELR+ motif, as well as CXCL1 and CXCL3 [29,30]. In general, CXCL2 is well known to be produced by inflammatory cells, and binds to CXCR2 to promote chemotaxis of neutrophils [29,30]. CXCL2 could also activate the STAT3 signalling pathway, which then regulates PD-L1 expression, in hepatocellular carcinoma cell lines [31]. Conditioned medium of primary cultured CAFs increased PD-L1 mRNA level in lung adenocarcinoma cell lines more than CXCL2 did. This difference might be attributed to the influence of other soluble factors present in conditioning medium of CAFs. Further examinations, such as long-term treatment or combination treatment of growth factors, chemokines, cytokines, exosomes, and extracellular matrix, are required to clarify the mechanisms of PD-L1 induction by CAFs in lung adenocarcinoma cells.

In this study, there were no differences between the results of cytokine array analysis of pFSC-1 and pFSC-2 conditioned media. However, they did have different effects on PD-L1 expression in PC-9, A549, and H1975 cells. Origins and functions of CAFs are well known as heterogeneous [2,4]; not all CAFs may have the same function as pFSC-1 or pFSC-2, and the influence of CAFs on PD-L1 expression may differ from each subset of CAFs. Therefore, the intratumoural heterogeneity of CAFs could subsequently induce heterogeneous expression of PD-L1 in adenocarcinoma tissues. Environmental factors are also considered to be important in the regulation of PD-L1 expression. For instance, the expression of α-SMA was significantly related to smoking history in this study. Some reports also demonstrated that smoking increased expression of α-SMA in fibroblasts [32]. Smoking might also enhance the functions of α-SMA including cytokine secretion in cancer tissue. In this study, results did demonstrate that soluble factors in conditioned medium collected from CAFs certainly influenced PD-L1 expression in lung adenocarcinoma cells. The expression of PD-L1 in cancer tissue is known to be heterogeneous in most cases, and strongly influenced by cancer tissue microenvironment factors, such as hypoxia, and cytokines including type I and type II interferons (IFNs) [14,28,33,34]. The correlation between carcinoma cells and its tissue microenvironment components represents the complicated network, and the influence of CAFs on PD-L1 expression in carcinoma cells may represent just one fraction. Therefore, we must consider the influence of other components of cancer microenvironments on PD-L1 expression in cancer cells and the status of CAFs. Previous reports demonstrated that CAFs influence the effects of anti-PD-1/-PD-L1 inhibitors in mouse models. In pancreatic ductal adenocarcinoma, FAP-positive CAFs suppressed the effects of anti-PD-L1 treatment through CXCL12/CXCR4 signalling [35]. Liu et al. [36] also reported that IL-6 secreted by CAFs suppressed anti-tumour immunity via impairing T-cell function, and inhibition of IL-6 enhanced the efficacy of anti-PD-L1 treatment in hepatocellular carcinoma mouse models. The results in this study indicated that CAFs might influence anti-PD-1/PD-L1 therapy, not only by suppressing tumour immunity, but rather by upregulating PD-L1 expression.

**Figure 6:**
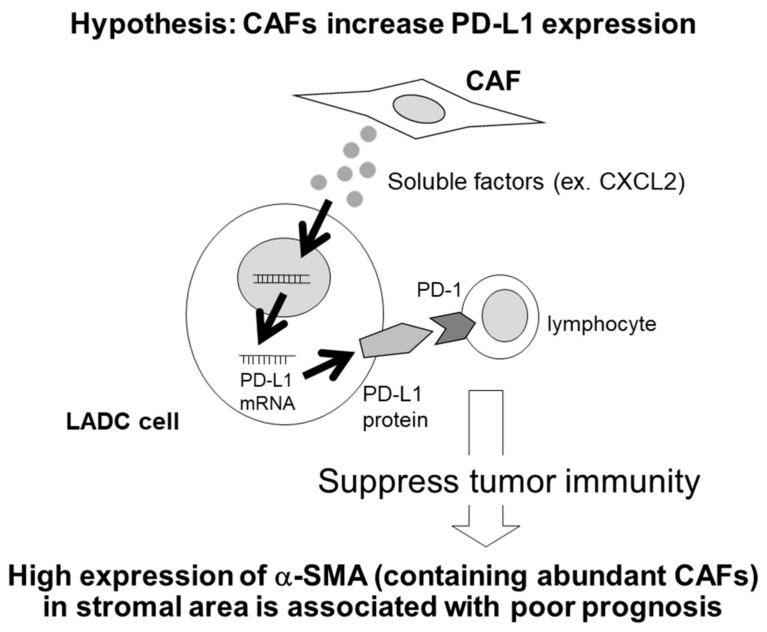
Proposed PD-L1 expression in interaction of cancer-associated fibroblasts (CAFs) and lung adenocarcinoma (LADC) cells. PD-L1 expression is induced by soluble factors such as CXCL2 derived from CAFs in the LADC cell. Interaction of PD-L1 with programmed death 1 (PD-1) contributes to the immune escape and poor prognosis of tumour cells.

The proposed PD-L1 expression patterns in the interaction between CAFs and lung adenocarcinoma cells are illustrated in Figure 6. In this study, CAFs, defined by α-SMA expression, did not relate to the infiltration of immune cells in lung adenocarcinoma cells. However, CAFs increased PD-L1 expression in lung adenocarcinoma cells through the secretion of soluble factors, such as CXCL2. Our results did indicate that CAFs could indirectly influence tumour immunity through increasing PD-L1 expression in lung adenocarcinoma cells.

## 4. Materials and Methods 

### 4.1. Patients

In this study, 27 patients (Cohort 1), who received EGFR-TKI therapy for recurrent lung adenocarcinoma, were first studied as a screening to elucidate the possible association between CAFs and tumor immunity in lung adenocarcinoma tissue (Cohort 1). We then studied 113 lung adenocarcinoma cases, including these 27 cases (Cohort 2). These cases were all retrieved from Tohoku University Hospital and Miyagi Cancer Centre between 2000 and 2008. All of these patients did not receive chemotherapy or radiation therapy prior to surgery. Clinicopathological characteristics of these patients were summarized in Table 1; Table 2. The Brinkman index was defined as the number of cigarettes smoked per day times smoking years. The specimens had all been fixed with 10% formalin and embedded in paraffin. Informed consent was obtained from each patient regarding the use of clinical records and tissue samples. This study was performed in accordance with the Declaration of Helsinki. The protocol for this study was approved by the Ethics Committee at the Tohoku University School of Medicine (2018-1-613), and the Ethics Committee at Miyagi Cancer Centre (No. 34).

### 4.2. Immunohistochemistry

For immunohistochemistry, we used the antibodies against the following proteins: α-SMA (dilution: 1/3000, Clone: 1A4, DAKO, Carpinteria, CA, USA), PD-L1 (Clone: SP263, Ventana Medical Systems, Tucson, AZ, USA), Ki-67 (dilution: 1/100, Clone: MIB-1, DAKO), CD3 (dilution: 1/500, Clone: F7.2.38, DAKO), CD4 (dilution: 1/1, Clone: 1F6, Nichirei bioscience, Tokyo, Japan), CD8 (dilution: 1/50, Clone: C8/144B, DAKO), Foxp3 (dilution: 1/100, Clone: 236A/E7, Abcam). We immunostained the sections with Histofine Kit (Nichirei bioscience, Tokyo, Japan) for α-SMA, Ki-67, CD3, CD4, CD8, and Foxp3. Antigen retrieval of Ki-67, CD3, CD8, and Foxp3 was performed by autoclaving the slides in citric acid for 5 min at 121 °C. Antigen retrieval of CD4 was performed by autoclaving the slides in target retrieval solution pH 9.0 (Nichirei) for 5 min at 121 °C. Antigen retrieval procedure was not employed for α-SMA.

Primary antibodies, except for PD-L1, were incubated overnight at 4 °C. After incubation with secondary antibody, immune complexes were detected with 3, 3-diaminobenzidine (DAB), and counterstained with hematoxylin. α-SMA immunoreactivity was detected in the cytoplasm of stromal cells. We evaluated the percentage of the stromal area of the tumour where positive α-SMA was detected in stromal fibroblasts in each case, according to previous reports [24,26,37,38]. Ki-67 labelling index (LI) was determined by counting 1000 tumour cells in the hot spots. Total numbers of positive lymphocytes for each marker (CD3, CD4, CD8, and Foxp3) were counted in four independent high-power microscopic fields (400×, 0.0625 mm^2^) [39].

Human PD-L1 antibody assay was optimized for use with Ventana OptiView DAB IHC Detection Kit (Ventana Medical Systems) on the BenchMark ULTRA platform autostainer (Ventana Medical Systems) [13]. A tumour was tentatively classified as PD-L1-positive if membrane staining was detected in ≥1% of the tumour cells [13,40]. Immunostaining of α-SMA and PD-L1 was performed on serial mirror tissue sections to examine their co-localization in the tumour.

### 4.3. Cell Lines

We used the lung adenocarcinoma cell lines A549 (EGFR wild-type), PC-9 (exon 19 deletion), and H1975 (L858R/T790M), obtained from American Type Culture Collection (Manassas, VA, USA). Primary CAFs, named pFSC-1 and pFSC-2, were isolated from human lung adenocarcinoma as described in previous studies [41]. Normal fibroblasts, OUS-11, were obtained from Japanese Collection of Research Bioresourse (Osaka, Japan). Cells were maintained under a humidified atmosphere of 5% CO_2_ at 37 °C in RPMI 1640 medium (Sigma Aldrich, St. Louis, MO, USA), containing 10% of fetal bovine serum (FBS; biosera, Boussens, France).

### 4.4. Immunocytochemistry

pFSC-1 and pFSC-2 were seeded on a Millicell EZ glass slide (Merck Millipore, Billerica, MA, USA), and incubated for 24 h. CAFs were fixed with 10% Formalin Neutral Buffer Solution (Wako pure chemical industries, Osaka, Japan). After blocking with rabbit serum, anti-α-SMA antibody was applied to the slide and incubated overnight at 4 °C. CAFs were visualized with DAB and stained with hematoxylin.

### 4.5. Conditioned Medium

The culture supernatants of pFSC-1 and pFSC-2 were collected as conditioned medium every 48–72 h, filtrated through Minisart NML syringe 0.8 μm pore filters (Sartorius, Göttingen, Germany), and stored at −80 °C. A549, PC-9, and H1975 cells were seeded onto 6-well plates at a density of 4 × 10^3^ cells/2 mL per well. After culture in 80% conditioned medium for 6 days, total protein or total RNA was extracted. RPMI 1640 medium containing FBS was used for control.

### 4.6. Quantitative RT-PCR

Total RNA was extracted from adenocarcinoma cell lines using TRIzol (Life Technologies, Carlsbad, CA, USA). RNA concentration was determined by Nano Drop one (Thermo Fischer Scientific, MA, USA). cDNA was synthesized from total RNA (1000 ng) using QuantiTect reverse transcriptional kit (Qiagen, Hilden, Germany), according to the manufacturer’s instructions. Quantitative RT-PCR was performed using Light cycler 96 (Roche). PD-L1 mRNA levels were normalized to RPL13A mRNA in the same sample.

The primer sequences were as follows: RPL13A (forward, 5′-CCT GGA GGA GAA GAG GAA AG-3′; reverse, 5′-TTG AGG ACC TCT GTG TAT TT-3′), PD-L1 (forward, 5′-CAA AGA ATT TTG GTT GTG GA-3′; reverse, 5′-AGC TTC TCC TCT CTC TTG GA-3′) [42], CXCL2 (forward, 5′-GGC AGA AAG CTT GTC TCA ACC C-3′; reverse, 5′-CTC CTT CAG GAA CAG CCA CCA A-3′) [43]. The primers were purchased from Nihon Gene Research Laboratories (Sendai, Japan).

### 4.7. Capillary Electrophoresis Immunoassay

Total cell protein was extracted using Mammalian Protein Extraction Reagent (Thermo Fischer Scientific), supplemented with 1% Halt Protease Inhibitor Cocktail (Pierce Biotechnology, Rockford, IL, USA). The supernatants were collected after centrifugation at 15,000 rpm at 4 °C for 5 min. Protein concentration was measured by Protein Assay Rapid Kit (Wako), according to the manufacturer’s instructions. Capillary electrophoresis immunoassay for detection of PD-L1 protein was employed by using Simple Western System Wes (ProteinSimple, California, USA). Protein samples and reagents (EZ Standard Pack 1, ProteinSimple) were loaded into the assay plate. The protein (ng/mL) was electrophoresed in capillary, which was filled with a stacking and a separation matrix (Jess/Wes 25-Capillary Cartridge, ProteinSimple). The proteins separated by the photoreactive binding reaction were immobilized on the inner wall of the capillary. Primary antibodies were as follows: Anti-PD-L1 XP monoclonal antibody (E1L3N, Cell Signaling Technologies, Danvers, MA, USA) at 1:100, or anti-β-actin monoclonal antibody (Sigma–Aldrich) at 1:1000. The target proteins were immunodetected with HRP-labeled secondary antibody and a chemiluminescent substrate (ProteinSimple). The data were analyzed using Compass software (ProteinSimple). 

### 4.8. Co-Culture System

The co-culture system was performed using a ThinCerts cell culture transparent membrane insert with 0.4 μm pores in 6-well plates (Greiner Bio-One, Kremsmünster, Austria). Both pFSC-1 and pFSC-2 were placed in the bottom chamber, with or without A549 cells, and PC-9 cells were placed in the upper chamber. After 72 h of co-culture, transwell chambers were removed, and the culture medium was replaced by FBS and phenol red-free medium. After 24 h, the conditioned media of fibroblasts with or without co-culture were collected.

### 4.9. Cytokine Analysis

We used Human Cytokine Antibody Array 5 (RayBiotech, Norcross, GA, USA) to identify cytokines secreted by fibroblasts. Cytokine antibody membranes were incubated for 5 h with 1 mL of fibroblast conditioned media with or without adenocarcinoma cell co-culture. Membranes were incubated overnight with biotin-conjugated anti-cytokine antibodies, and then developed with horseradish peroxidase–streptavidin and chemiluminescence. The images were visualized using Molecular Imager ChemiDOC XRS+ (Bio-Rad, Hercules, CA), and quantified by Image Lab Software (Bio-Rad).

Recombinant human CXCL2, IL-8, and TNFRSF11B (osteoprotegerin, OPG) were purchased from BioLegend (San Diego, CA, USA). CXCL2, IL-8, and OPG were added into the medium. PD-L1 mRNA and protein expression were examined as described. We preliminarily examined the optimal concentration of CXCL2 in each cell line, and found that 100 ng/mL CXCL2 significantly suppressed the viability of PC-9. Therefore, we employed 10 ng/mL CXCL2 for PD-L1 induction in both PC-9 and H1975 cells. Otherwise, in A549, 100 ng/mL CXCL2 did not affect cell survival.

### 4.10. Statistical Analysis

All statistical analyses were performed using JMP Pro 13.0.0 (SAS Institute, Japan, Tokyo). Statistical differences between the two groups of immunohistochemical analysis were evaluated by Wilcoxon signed-rank test, Fisher’s exact test, Chi-squared test, or Spearman’s rank correlation coefficient. Five-year overall survival curves were generated according to the Kaplan–Meier method, and the statistical significance was calculated using the log-rank test. The Cox proportional hazards model was used for multivariate analysis. Results of in vitro study were demonstrated as mean ± SD. Statistical analyses of in vitro study were evaluated by t-test. Statistical significance was defined as *p* < 0.05 in this study.

## 5. Conclusions

The expression of α-SMA, a common marker of CAFs, in cancer stroma was associated with PD-L1 expression in adenocarcinoma cells. CAFs increased PD-L1 expression in lung adenocarcinoma cells through the secretion of soluble factors, including CXCL2. Our results indicated that CAFs might influence tumour immunity through increasing PD-L1 expression in lung adenocarcinoma cells.

## Figures and Tables

**Table 1:** Association between α-SMA status and clinicopathological parameters in lung adenocarcinoma cases (Cohort 1).

		Total	α-SMA	α-SMA	*p*-Value
			High	Low	
Age	median	64	63	64	0.9803
(years)	max	80	80	74	
	min	34	34	46	
Sex	male	8	6	2	
	female	19	10	9	
Smoking	smoker	6	5	1	0.3497
	never	21	11	10	
Brinkman index	median	0†	0†	0†	0.1646
	max	1200	1200	700	
	min	0	0	0	
Size of tumor	median	28	27	30.5	0.9069
(mm)	max	80	80	40	
	min	13	15	13	
EGFR	exon 19 del	12	3	9	0.3172
mutation	exon 21 L858R	11	6	5	
	G719X, S768I	1	1	0	
	ex20 Ins	1	0	1	
	unknown	2	1	1	
Response to	CR	1	1	0	0.7354
EGFR-TKI	PR	17	10	7	
	SD	8	4	4	
	PD	1	1	0	
Ki-67 LI of carcinoma cells (%)	median	12.3	13.1	10.9	0.5053
	max	58.8	58.8	32	
	min	3.3	3.3	4.7	
pStage	I	9	2	7	0.0263*
	II	5	4	1	
	III	10	8	2	
	IV	3	2	1	
pT	1	13	7	6	0.6146
	2	11	7	4	
	3	1	1	0	
	4	2	1	1	
pN	0	13	5	8	0.0514#
	1	5	4	1	
	2	9	7	2	
	3	0	0	0	
cM	0	24	14	10	1.000
	1	3	2	1	
PD-L1	positive	5	5	0	0.0598#
	negative	22	11	11	
CD3	median	766	758	767	0.941
	max	1656	1656	1022	
	min	404	404	462	
CD4	median	697	693.5	697	0.2669
	max	1486	1486	806	
	min	263	303	263	
CD8	median	416	432.5	399	0.7484
	max	611	599	611	
	min	177	209	177	
Foxp3	median	518	110.5	111	0.9214
	max	111	518	249	
	min	15	32	15	
Foxp3/CD8	median	0.3	0.28	0.31	0.941
	max	1	1	0.89	
	min	0.03	0.12	0.03	
CD8/CD4	median	0.67	0.61	0.7	0.4443
	max	1.3	1.03	1.3	
	min	0.26	0.33	0.26	

* *p*-value < 0.05, #0.05 ≤ *p*-value < 0.1, †The great majority of the patients were never-smokers in our present study. EGFR: epidermal growth factor receptor, Ki-67 LI: Ki-67 labeling index, PD-L1: programmed death ligand 1, CR: complete response, PR: partial response, SD: stable disease, PD: progressive disease.

**Table 2:** Association between α-SMA status and clinicopathological parameters in 113 lung adenocarcinoma cases (cohort 2).

		Total	α-SMA	α-SMA	*p*-Value
			High	Low	
Age	median	66	64.5	70	0.0872
(years)	max	82	80	82	
	min	30	30	46	
Sex	male	58	37	21	0.0241*
	female	55	23	32	
Smoking	smoker	61	38	23	0.0391*
	never	52	22	30	
Brinkman index	median	240	520	0†	0.0131*
	max	1920	1840	1920	
	min	0	0	0	
Size of tumor	median	25	24.5	27	0.2570
(mm)	max	80	80	70	
	min	10	10	10	
pStage	I	68	29	39	0.0022*
	II	13	7	6	
	III	24	17	7	
	IV	8	7	1	
pT	1	65	34	31	0.7978
	2	36	19	17	
	3	3	2	1	
	4	9	5	4	
pN	0	85	38	47	0.0015*
	1	8	6	2	
	2	19	15	4	
	3	1	1	0	
cM	0	105	53	52	0.0648#
	1	8	7	1	
PD-L1	positive	17	17	0	<0.0001*
	negative	96	43	53	

* *p*-value < 0.05, #0.05 ≤ *p*-value < 0.1, †The great majority of the patients were never-smokers in our present study.

## Supplementary Materials

The following are available online at https://www.mdpi.com/2072-6694/11/9/1257/s1
