# Supplementary material for: PD-L1 Induction by Cancer-Associated Fibroblast-Derived Factors in Lung Adenocarcinoma Cells
Source: Cancers (Basel). 2019 Aug 27;11(9):1257. doi: 10.3390/cancers11091257 (PMC6770125; doi:10.3390/cancers11091257)
Supplement: Supplementary file 1 [file cancers-11-01257-s001.zip › Supplementary Info File 20190803.docx]

Supplemental information for

**PD-L1 induction by cancer-associated fibroblast-derived factors in lung adenocarcinoma cells**

Chihiro Inoue^1^, Yasuhiro Miki^2^, Ryoko Saito^1^, Shuko Hata^3^, Jiro Abe^4^, Ikuro Sato^5^, Yoshinori Okada^6^, Hironobu Sasano^1^

^1^ Department of Anatomic Pathology, Tohoku University Graduate School of Medicine, Sendai, Miyagi, Japan

^2^ Department of Disaster Obstetrics and Gynecology, International Research Institute of Disaster Science, Tohoku University, Sendai, Miyagi, Japan

^3^ Division of Pathology, Faculty of Medicine, Tohoku Medical and Pharmaceutical University, Sendai, Miyagi, Japan

^4^ Department of Thoracic Surgery, Miyagi Cancer Centre, Natori, Miyagi, Japan.

^5^ Department of Pathology, Miyagi Cancer Centre, Natori, Miyagi, Japan

^6^ Department of Thoracic Surgery, Institute of Development, Aging and Cancer, Tohoku University, Sendai, Miyagi, Japan


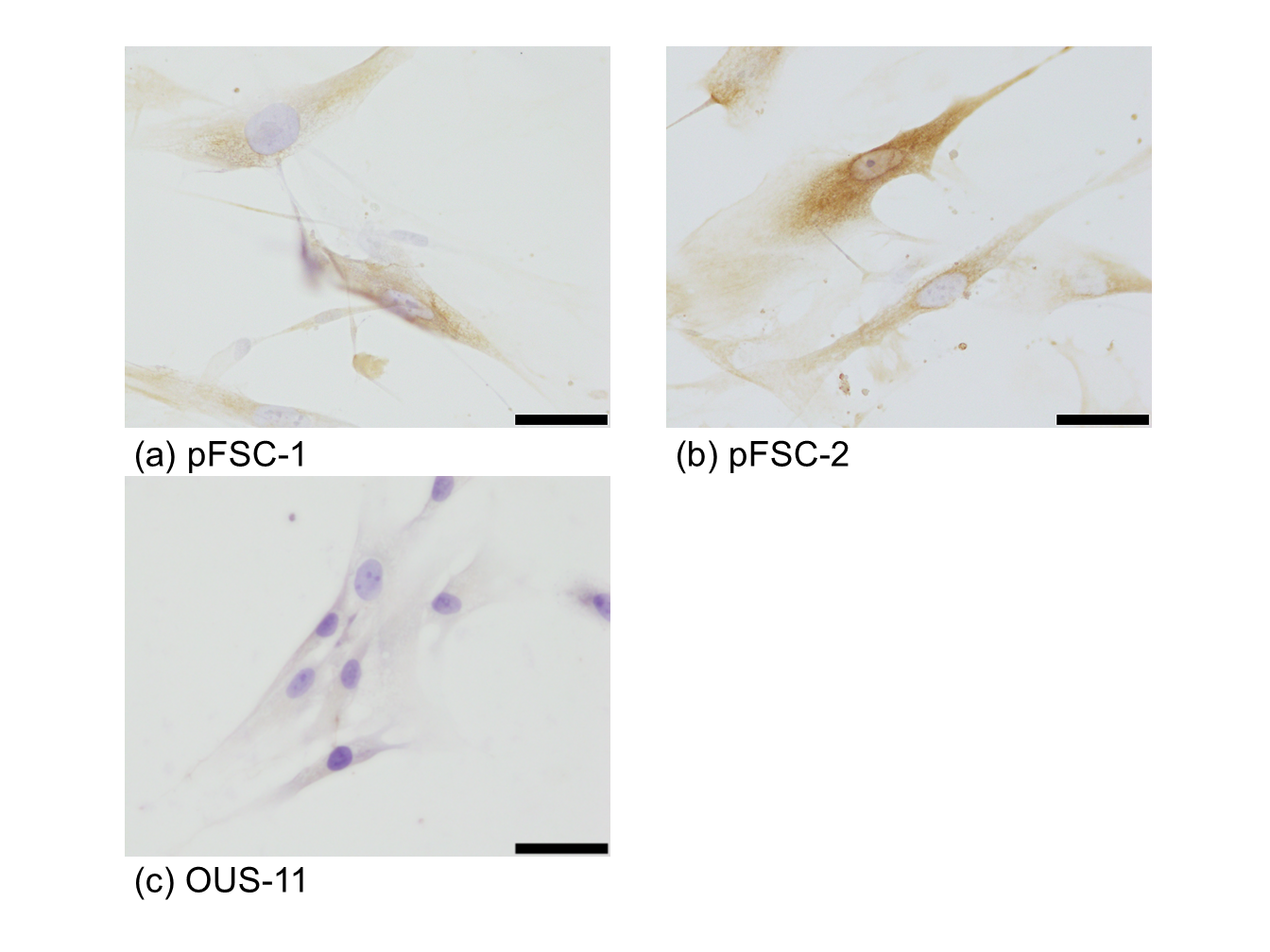


**Supplementary Figure S1**

Immunocytochemistry of α-SMA. (a) pFSC-1 and (b) pFSC-2 were α-SMA-positive, whereas (c) OUS-11 was α-SMA-negative. Bar=40 μm.


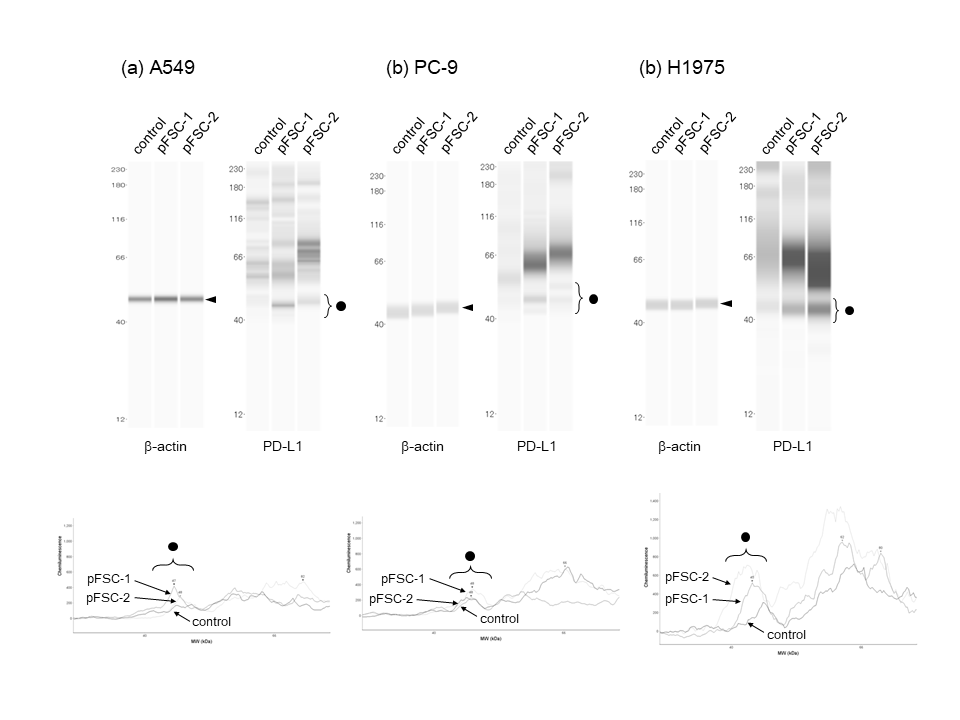


**Supplementary Figure S2**

Electropherograms and lane views of capillary electrophoresis immunoassay for detection of PD-L1 protein.


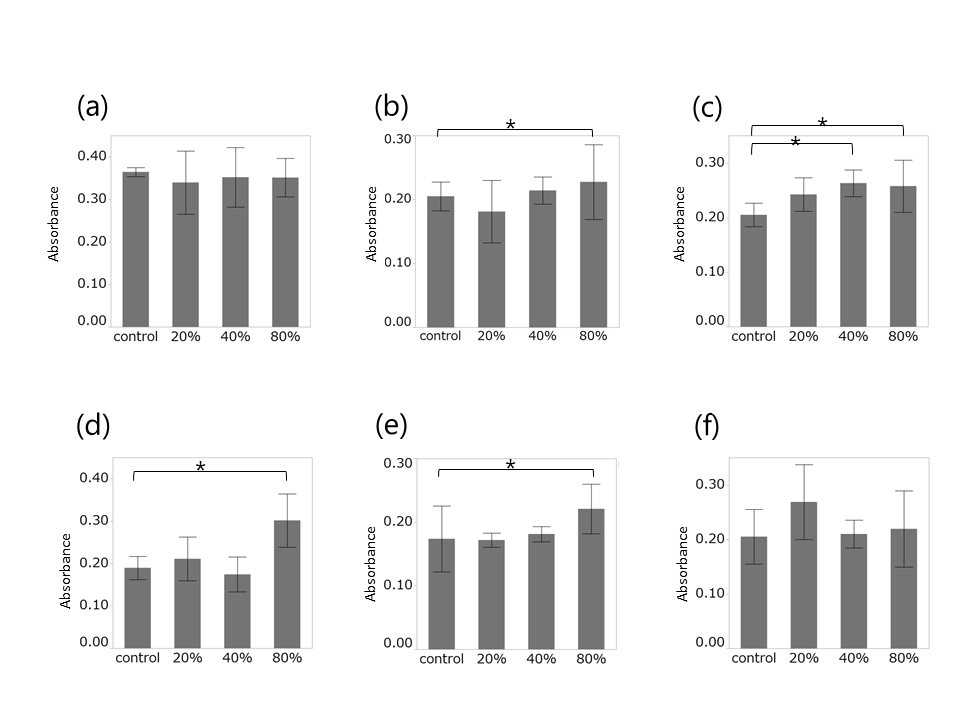


**Supplementary Figure S3**

Results of proliferation assay using conditioned medium of pFSC-1 [(a) A549, (b) PC-9, (c) H1975] and pFSC-2 [(d) A549, (e) PC-9, (f) H1975].

A549, PC-9, and H1975 cells were seeded onto 96-well plates at a density of 4×10^4^ cells/100 μL per well. After culture in 0 (as control), 20, 40, and 80% conditioned medium for 48 hours. We evaluated cell proliferation using the WST-8 colorimetric assay (Cell Counting Kit-8; Dojindo Laboratories, Kumamoto, Japan). The absorbance was measured at 450 nm (background absorbance at 650 nm subtracted) using iMark Microplate Reader (Bio-Rad, Hercules, CA).

N=6, *p-value<0.05.


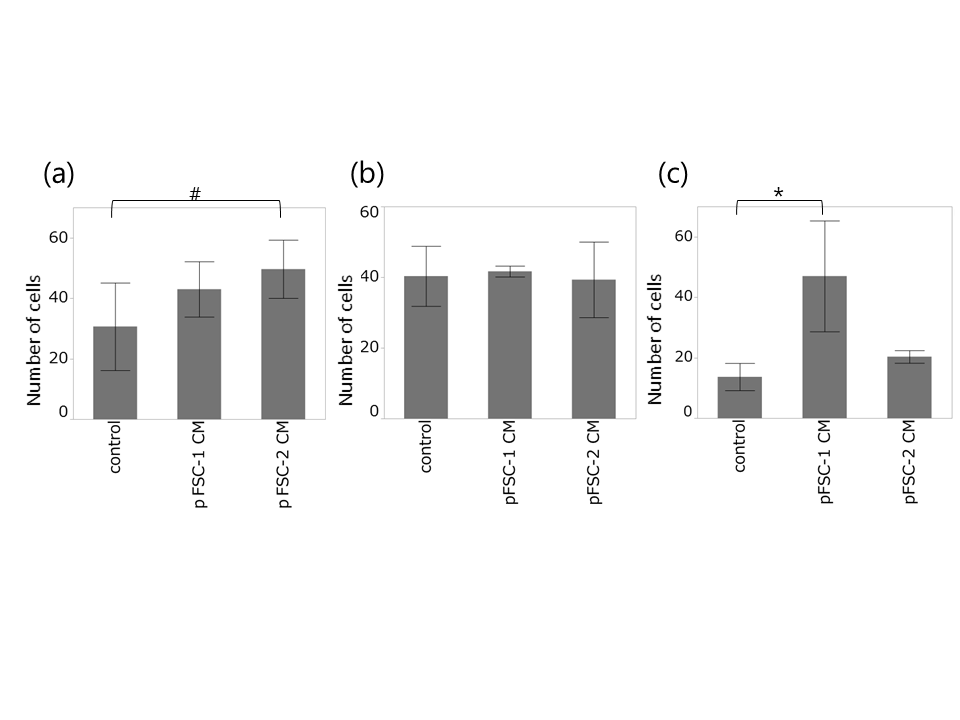


**Supplementary Figure S4**

Results of migration assay. (a) A549, (b) PC-9, (c) H1975.

A549, PC-9, and H1975 cells were seeded onto ThinCerts cell culture transparent membrane insert with 8.0 μm pore (Greiner Bio-One, Kremsmünster, Austria) at a density of 4×10^4^ cells/200 μL per insert. After incubation with or without 80% conditioned medium (CM) of pFSC-1/pFSC-2 for 24 hours, the cells on the upper surface of the membrane were mechanically removed with cotton swabs. The migrated cells were fixed in methanol and stained with hematoxylin and the membranes were mounted on glass slides. We counted the number of cells at five fields (×200) randomly selected.

N=3, *p-value<0.05, #0.05≤p-value<0.1
